# From Waste to Resource: Chemical Characterization of Olive Oil Industry By-Products for Sustainable Applications

**DOI:** 10.3390/molecules30153212

**Published:** 2025-07-31

**Authors:** Maria de Lurdes Roque, Claudia Botelho, Ana Novo Barros

**Affiliations:** 1 Centre for the Research and Technology of Argo-Environmental and Biological Sciences (CITAB), Institute for Innovation, Capacity Building and Sustainability of Agri-Food Production (Inov4Agro), University of Trás-os-Montes and Alto Douro (UTAD), Quinta de Prados, 5000-801 Vila Real, Portugal; marialroque39@gmail.com; 2 Centro de Estudos de Ciência Animal (CECA), Instituto de Ciências, Tecnologias e Agroambiente da Universidade do Porto (ICETA), Rua D. Manuel II, Apartado 55142, 4051-401 Porto, Portugal; claudiabotelho@ceb.uminho.pt; 3 Associate Laboratory for Animal and Veterinary Science (AL4AnimalS), 1300-477 Lisbon, Portugal

**Keywords:** circular economy, food waste valorization, phenolic compounds, antioxidant capacity, olive leaves, olive branches, olive stones, olive seeds, HPLC-PDA

## Abstract

The olive oil industry, a key component of Southern Europe’s agricultural sector, generates large amounts of by-products during processing, including olive leaves, branches, stones, and seeds. In the context of growing environmental concerns and limited natural resources—particularly in the Mediterranean regions—there is increasing interest in circular economy approaches that promote the valorization of agricultural residues. These by-products are rich in bioactive compounds, particularly phenolics such as oleuropein and hydroxytyrosol, which are well known for their antioxidant and anti-inflammatory activities. This study aimed to evaluate the phenolic content and antioxidant capacity of by-products from three olive cultivars using high-performance liquid chromatography with photodiode array detection (HPLC–PDA) and mass spectrometry (MS). The leaves and seeds, particularly from the “Cobrança” and a non-identified variety, presented the highest antioxidant activity, as well as the highest concentration of phenolic compounds, demonstrating once again the direct relationship between these two parameters. The identification of the compounds present demonstrated that the leaves and branches have a high diversity of phenolic compounds, particularly secoiridoids, flavonoids, phenylpropanoids, phenylethanoids, and lignans. An inverse relationship was observed between the chlorophyll and carotenoid content and the antioxidant activity, suggesting that phenolic compounds, rather than pigments, are the major contributors to antioxidant properties. Therefore, the by-products of the olive oil industry are a valuable source of sustainable bioactive compounds for distinct industrial sectors, such as the food, nutraceutical, and pharmaceutical industries, aligning with the European strategies for resource efficiency and waste reduction in the agri-food industries.

## 1. Introduction

The scarcity of food resources remains a major global concern, particularly in the Mediterranean regions, where intensive agricultural practices and excessive water consumption threaten ecosystem sustainability [1,2,3]. In response to these challenges, the European Union has promoted circular economy strategies that encourage the reuse and recovery of agricultural by-products, aiming to reduce waste and its associated environmental impacts [4,5].

The olive oil industry, a cornerstone of southern Europe’s agricultural economy, produces substantial quantities of solid and liquid waste during olive processing. These by-products—including leaves, branches, stones, and seeds—hold significant potential for valorization across diverse sectors, such as biofuel production, animal and human nutrition, cosmetics, and pharmaceuticals, as well as for the extraction of bioactive compounds with recognized health benefits [3,6].

The type and quantity of waste generated vary according to the oil extraction method employed. Traditional pressing and two- or three-phase centrifugation differ in terms of efficiency, resource consumption, and waste output. Traditional pressing produces considerable amounts of wastewater, whereas three-phase centrifugation allows for greater automation and improved oil quality but requires high water and energy input. Conversely, two-phase centrifugation minimizes liquid effluent production but yields moist by-products that are more challenging to manage [3,6,7].

Among the main by-products, olive leaves—collected during pruning and harvesting—are rich in phenolic compounds, such as oleuropein and hydroxytyrosol, known for their antioxidant, anti-inflammatory, and antimicrobial properties [8,9]. These bioactive compounds have also been associated with improved blood glucose control and insulin sensitivity, making them promising candidates for applications in the food, cosmetics, and pharmaceutical industries [10,11].

Olive branches have likewise garnered attention for their bioactive potential. They contain phenolics and flavonoids, including oleuropein, hydroxytyrosol, tyrosol, and verbascoside, compounds of interest for cosmetic applications. Additionally, they represent a viable biomass resource for bioenergy production [12,13].

The olive stone—the woody endocarp—can constitute up to 30% of the fruit’s total weight [14]. Composed mainly of cellulose, hemicellulose, and lignin, its rigid structure hinders the extraction of phenolic compounds, such as oleuropein and hydroxytyrosol, limiting its use in food and pharmaceutical applications [14,15,16]. Nonetheless, it has been successfully used in the production of energy, activated carbon, and cosmetic ingredients [15,17].

The olive seed has gained attention due to its valuable composition and wide range of applications. It contains proteins and lipids, particularly oleic acid (58.4–73.6%) and linoleic acid (17.1–24.2%) [14,18,19]. In addition, it is rich in dietary fiber (approximately 47% of its dry weight), essential macro- and micronutrients, and phenolic compounds, such as salidroside, nüzhenide, hydroxytyrosol, nüzhenide 11-methyl oleoside, oleuropein, tyrosol, and desmethyloleuropein, all of which exhibit antioxidant activity [20,21,22]. These characteristics make it a promising raw material for the production of activated carbon, animal feed additives, fertilizers, and solid fuels [23,24,25]. In addition to phenolic compounds, some olive by-products—particularly leaves and branches—contain significant levels of natural pigments, such as chlorophylls and carotenoids, which may also contribute to their antioxidant capacity. Therefore, the quantification of these pigments was included in this study to better understand their potential role and contribution to the overall bioactivity of these matrices [26,27].

Portugal is among the leading olive oil producers in the European Union, boasting around 65 olive varieties. The most significant varieties for olive oil production are ‘Cobrançosa’ and ‘Galega Vulgar’, both of which are protected under PDO (Protected Designation of Origin) status, ensuring product authenticity and quality [28,29].

The objective of this study is to evaluate the phenolic content and antioxidant capacity of different by-products derived from the processing of three olive cultivars. Analyses were conducted using high-performance liquid chromatography coupled with photodiode array detection (HPLC–PDA) and mass spectrometry (MS) to identify the specific compounds responsible for the antioxidant activity. This work aims to support the valorization of olive by-products and foster enhanced sustainability within the olive oil sector.

## 2. Results and Discussion

### 2.1. Phenolic Content

The phenolic content of the leaf, seed, stone, and branch from ‘Cobrançosa’, ‘Galega’, and NI cultivars was assessed, as presented in Table 1.

The results obtained reveal marked differences in the content of phenolic compounds, *ortho*-diphenols, and flavonoids among the various olive tree by-products and cultivars analyzed.

The highest total phenolic content was found in the seeds of the ‘Cobrançosa’ variety (86.116 ± 0.336 mg GA/g DW), followed by the non-specified (NI) variety (78.032 ± 2.087 mg GA/g DW). These values are considerably higher than those reported by Gouvinhas et al. [21] for ‘Cobrançosa’ seeds (11.90 ± 1.56 mg GA/g DW). This discrepancy may be attributed to the differences in the extraction methods employed, as well as to regional variability, since although the samples belong to the same cultivar, they originate from distinct areas in Portugal. Similar values to those found in this study were reported by Morcy et al. [30], who observed a total phenolic content of 85.12 ± 1.33 mg GA/g DW in the skin of sesame seeds. Conversely, the lowest values were recorded in the stones of the ‘Galega’ (6.534 ± 0.614 mg GA/g DW) and NI (9.310 ± 0.489 mg GA/g DW) varieties. These are comparable to those reported by Djemaa-Landri et al. [31] for olive stones from seven Algerian cultivars (ranging between 5.14 ± 0.16 and 7.23 ± 0.08 mg GA/g DW). Similar findings were also reported for two Tunisian varieties: 212.19 ± 6.56 mg GA/100 g DW in the ‘Zarrazi’ cultivar and 761.83 ± 1.97 mg GA/100 g DW in ‘Chemlali’, although it should be noted that in these studies, the stones contained seeds, and the cultivars differ from those assessed in the present work.

Despite the higher values observed in the leaf of the NI variety (66.147 ± 4.87 mg GA/g DW), ‘Cobrançosa’ consistently exhibited higher phenolic contents across the remaining by-products. These differences may be related to phenolic profile variations between cultivars at different ripening stages, as demonstrated by Ferro et al. [32]. Comparable values to those found for the NI variety were also observed in the leaf of the northern Iranian cultivar ‘Mary’ (62.24 ± 0.012 mg GA/g DW).

Regarding *ortho*-diphenols, the highest concentration was found in the leaf of the NI variety (89.301 ± 4.463 mg GA/g DW), followed by ‘Cobrançosa’ (78.177 ± 0.681 mg GA/g DW). Few studies have specifically characterized *ortho-*diphenol content, but those by Tekaya et al. [33] and Abdeljelil et al. [34] reported significantly lower values, likely reflecting varietal differences. The lowest values were again observed in the stones of NI (9.389 ± 0.446 mg GA/g DW) and ‘Galega’ (6.882 ± 0.545 mg GA/g DW). Paié-Ribeiro et al. [7] similarly reported *ortho*-diphenol contents in pomace from a two-phase process (9.32 ± 0.60 mg GA/g DW). The NI variety showed significantly higher *ortho*-diphenol levels in the leaves and seeds (89.301 ± 4.463 and 42.857 ± 0.158 mg GA/g DW, respectively), while ‘Cobrançosa’ stood out in the stone and branch by-products (11.696 ± 1.351 and 35.209 ± 2.396 mg GA/g DW, respectively). Gouvinhas et al. [21] also analyzed the *ortho*-diphenols in ‘Cobrançosa’ and ‘Galega’ seeds but reported lower levels (4.69 ± 0.16 and 7.05 ± 0.44 mg GA/g DW, respectively), possibly due to methodological or regional differences.

As for flavonoids, the highest values were found in the leaves of the NI variety (54.297 ± 5.073 mg CAT/g DW), followed by ‘Cobrançosa’ (39.119 ± 1.321 mg CAT/g DW). Oliveira et al. [35] reported similar values in ‘Cobrançosa’ leaves (30.40 ± 0.80 mg caffeic acid equivalents [ACE]/g DW), and comparable concentrations were observed in the ‘Verdeal’ cultivar (53.70 ± 5.20 mg mg epicathechin equivalents [ACE]/g DW). The branches of the ‘Cobrançosa’ cultivar also displayed a high flavonoid content (51.715 ± 5.389 mg CAT/g DW), significantly greater than that of ‘Galega’ (35.521 ± 1.556 mg CAT/g DW). In contrast, Benčić et al. [36] reported much lower values (3.64 ± 0.03 and 2.87 ± 0.32 mg EC/g DW for samples stored at room temperature and 4 °C, respectively), likely due to differences in cultivars, extraction solvents, and environmental conditions.

Among the other by-products, the highest flavonoid levels were found in the stones (21.180 ± 2.706 mg CAT/g DW) and seeds (10.849 ± 0.641 mg CAT/g DW) of ‘Cobrançosa’. Djemaa-Landri et al. [31] reported markedly lower flavonoid contents for the stones of Algerian varieties (ranging from 0.06 ± 0.09 to 0.36 ± 0.01 mg quercetin equivalents [QE]/g DW), likely reflecting genetic and climatic differences. Gouvinhas et al. [21] also found lower flavonoid levels in ‘Cobrançosa’ seeds (1.50 ± 0.20 mg CAT/g DW), which could be explained by regional and agronomic variations, given that phenolic composition is strongly influenced by factors such as soil, climate, and cultivation practices.

Overall, the ‘Cobrançosa’ cultivar exhibited the highest levels of phenolic compounds and flavonoids in comparison with ‘Galega’. Leaves and branches were the most phenolic-rich by-products, while stones generally presented the lowest concentrations.

### 2.2. Antioxidant Capacity

The antioxidant capacity of the leaves, seeds, stones, and branches from the ‘Cobrançosa’, ‘Galega’, and NI cultivars was assessed, as presented in Table 2.

In view of the results obtained, there were significant variations in the antioxidant capacity between the different parts of the olive tree and between the varieties analyzed. Overall, the leaves showed the highest antioxidant capacity values, while the stones consistently exhibited the lowest values. Although the branches also presented high antioxidant activity, in some cases the seeds exhibited values statistically similar to or higher than the branches, depending on the variety and the method used. With regard to the ABTS method, the leaves of the unknown variety (NI) showed the highest antioxidant capacity values (0.323 ± 0.056 mmol Trolox g^−1^ DW), followed by ‘Cobrançosa’ (0.286 ± 0.007 mmol Trolox g^−1^ DW). Previous studies, like Ronca et al. [10], have reported similar values for olive leaves from various regions of Portugal, between 0.19 ± 0.020 and 0.51 ± 0.05 mmol Trolox/g DW for ABTS and between 0.06 ± 0.01 and 0.19 ± 0.01 mmol Trolox/g DW for DPPH. For the DPPH and FRAP methods, the NI leaf variety also produced the highest values (0.206 ± 0.009 and 0.676 ± 0.036 mmol Trolox g^−1^ DW, respectively). Ghasemi et al. [37] reported a similar antioxidant activity value of FRAP in the cultivar ‘Shenge’ (614.19 ± 0.004 µmol Fe^2+^/g DW). In contrast, the ‘Galega’ variety showed the lowest values in all the methods tested (0.201 ± 0.009 µmol Trolox g^−1^ DW for ABTS, 0.076 ± 0.002 mmol Trolox g^−1^ DW for DPPH, and 0.281 ± 0.007 mmol Trolox g^−1^ DW for FRAP). To date, there have been no studies comparing the leaves of the ‘Galega’ variety with other varieties; however, the results of this study suggest that ‘Galega’ may have a lower content of phenolic compounds than other cultivars, which could explain the lower antioxidant activity observed.

For the seeds, antioxidant activity remained relatively homogeneous across cultivars. In the ABTS assay, the ‘Cobrançosa’ and the NI varieties presented similar values (0.251 ± 0.003 and 0.247 ± 0.005 mmol Trolox g^−1^ DW, respectively), while ‘Galega’ showed a slightly lower value (0.233 ± 0.003 mmol Trolox g^−1^ DW). Although no statistically significant differences were found in the DPPH assay, ‘Cobrançosa’ exhibited the lowest mean value (0.051 ± 0.043 mmol Trolox g^−1^ DW), possibly due to high variability in the data. With the FRAP method, ‘Cobrançosa’ and ‘Galega’ exhibited similar values (0.164 ± 0.002 and 0.160 ± 0.003 mmol Trolox g^−1^ DW, respectively), while the NI variety had slightly higher activity (0.186 ± 0.003 mmol Trolox g^−1^ DW). Previous studies have reported lower values than those obtained in this work. Gouvihas et al. [21] observed lower values for the DPPH and ABTS methods; however, the extraction method was different, and the varieties are from a different region. Similarly, Falcinelli et al. [38] reported antioxidant activity in olive seeds ranging from 2 to 10 µmol Trolox g^−1^ DW using the FRAP method, which may reflect differences in cultivar and methodological approach.

The stones exhibited the lowest antioxidant capacity among all the by-products analyzed. The ‘Cobrançosa’ variety presented the highest values within this group, notably for the FRAP method (0.127 ± 0.012 mmol Trolox g^−1^ DW), followed by the NI variety (0.088 ± 0.016 mmol Trolox g^−1^ DW), and finally ‘Galega’ (0.067 ± 0.007 mmol Trolox g^−1^ DW). The ABTS and DPPH values were similarly low across all cultivars. Djemaa-Landri et al. [29] reported the DPPH values between 9.93 ± 0.28 and 32.87 ± 1.31 mg TE/g DW in stones from different olive cultivars, which are similar to the values described here. Currently, there are no data in the literature on the antioxidant activity of olive stones using the FRAP method, but Uribe et al. [38] evaluated pomace (a mixture that includes pulp and stone) and obtained a value of 100 µmol TE/g DW using FRAP. The similar antioxidant activity in their results may contribute to the higher antioxidant capacity compared with the pulp.

In the case of the branches, ‘Cobrançosa’ exhibited the highest antioxidant capacity, with values of 0.240 ± 0.025 mmol Trolox g^−1^ DW (DPPH) and 0.242 ± 0.006 mmol Trolox g^−1^ DW (FRAP), while the lowest values were found in the NI variety (ABTS: 0.174 ± 0.048; DPPH: 0.151 ± 0.041; and FRAP: 0.126 ± 0.021 mmol Trolox g^−1^ DW). Currently, few studies have addressed the antioxidant potential of olive branches. Uribe and Bruno [39,40] reported higher antioxidant activities in olive wood using DPPH and FRAP (759 and 350 µmol Trolox g^−1^ DW, respectively), likely due to the use of different extraction solvents and wood samples from Italian cultivars. Additionally, Gullón et al. [41] obtained 180 µmol Trolox g^−1^ DW for olive pruning residues using the ABTS method, which is similar to the value obtained in ‘Galega’. The differences in solvent polarity (e.g., 50% ethanol) and sample matrix (wood vs. branch vs. pruning residue) could explain the variation in results.

In general, the leaf was the by-product with the highest antioxidant capacity, followed in some cases by the seed or branch, depending on the method and variety, while the stone consistently exhibited the lowest activity; ‘Cobrançosa’ showed greater antioxidant activity compared to ‘Galega’. These results suggest that the leaves and branches may be promising sources of natural antioxidants for potential applications in the food, cosmetic, or pharmaceutical industries.

#### Multivariate and Regression Analyses Reveal the Phenolic Drivers of Antioxidant Capacity in Olive Oil By-Products

To comprehensively explore the biochemical complexity and antioxidant potential of olive oil by-products, we employed an integrated multivariate statistical approach that included hierarchical clustering (heatmap), principal component analysis (PCA), partial least squares (PLS) regression, and variable importance in projection (VIP) scoring. These analyses allowed us to identify the phenolic compounds most responsible for the antioxidant behavior and to assess the impact of tissue type and cultivar origin on phytochemical profiles.

**Figure 1:**
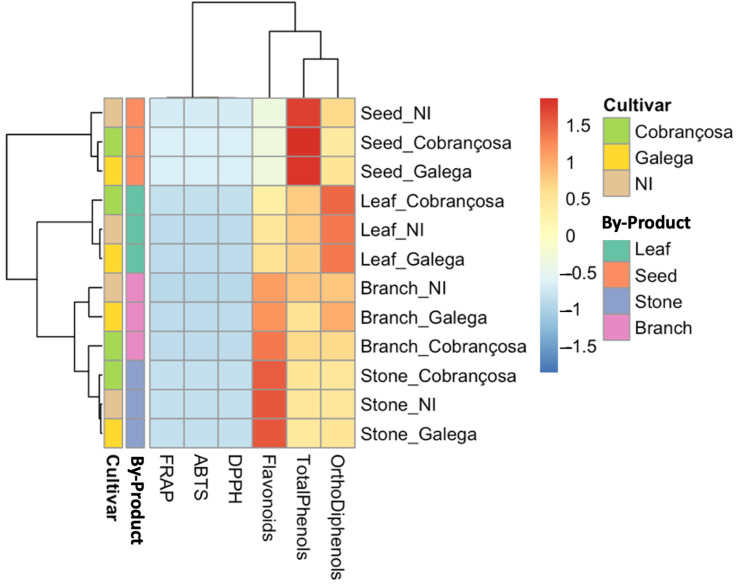
Heatmap of standardized phenolic content and antioxidant capacity in olive oil by-products from three cultivars (NI, ‘Cobrançosa’, and ‘Galega’). The hierarchical clustering was performed using Z-score normalized values for total phenols, *ortho*-diphenols, flavonoids, ABTS, DPPH, and FRAP. Samples are grouped by cultivar and by-product (leaf, seed, branch, stone), revealing distinct biochemical profiles. Higher values are represented in red and lower values in blue. ‘Cobrançosa’ and NI samples, especially from seeds and leaves, clustered separately based on their enriched phenolic and antioxidant profiles.

Figure 1 presents a heatmap generated using Z-score-normalized values for total phenols, *ortho*-diphenols, flavonoids, and antioxidant capacity, as measured by ABTS, DPPH, and FRAP. Clear clustering patterns emerged based on both tissue type and cultivar. Samples from leaves and seeds of the ‘Cobrançosa’ and NI cultivars consistently appeared in a high-intensity cluster, reflecting enriched phenolic profiles and elevated antioxidant activity. These tissues are known to be particularly rich in bioactive compounds, such as oleuropein, hydroxytyrosol, and verbascoside, and their bioactivity has been well-documented in previous studies [42,43,44,45].

In contrast, samples from stones, especially from the ‘Galega’ cultivar, clustered at the lower end of the biochemical spectrum, consistent with their low extractable phenolic content and limited antioxidant potential. Branches displayed an intermediate pattern, with ‘Cobrançosa’ branches demonstrating notably stronger antioxidant values than those from ‘Galega’, suggesting that this underutilized tissue may hold promise for functional applications.

**Figure 2:**
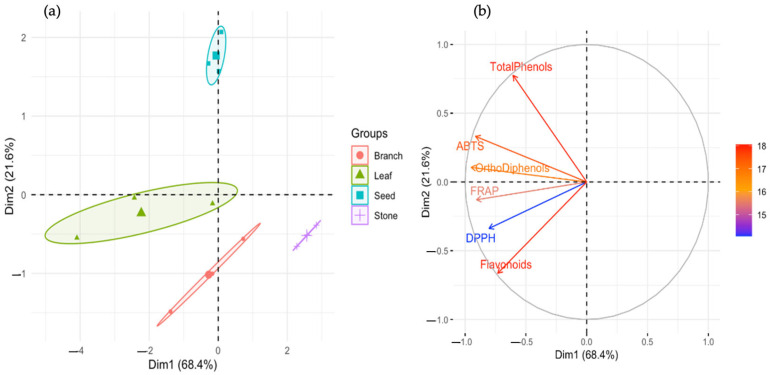
Principal component analysis (PCA) of phenolic content and antioxidant capacity across olive oil by-products. (**a**) PCA score plot showing sample clustering by by-product type (leaf, seed, branch, stone) and cultivar (NI, ‘Cobrançosa’, ‘Galega’). PC1 (68.4%) and PC2 (21.6%) explain most of the data variance. Samples with high phenolic content and antioxidant activity (e.g., NI seeds and Cobrançosa leaves) cluster separately from low-phenolic groups (e.g., stones). (**b**) PCA loading plot (variable contribution) illustrating the influence of each variable on PC1 and PC2. Total phenols and *ortho*-diphenols contribute strongly to PC1, while flavonoids and ABTS drive variation along PC2. Arrow length and direction indicate variable importance and correlation with sample grouping.

Principal component analysis further confirmed these distinctions. As shown in Figure 2a, the first two components explained 68.4% and 21.6% of the total variance, respectively, totaling 90% of the dataset’s variability. Samples clustered according to both by-product type and cultivar, with leaf and seed samples, particularly from ‘Cobrançosa’ and NI, grouping together in the positive quadrant of PC1, which was strongly associated with antioxidant capacity and phenolic content. Stone samples were located on the negative end of PC1, reinforcing their biochemical underperformance. In Figure 2b, the PCA loading plot demonstrated that total phenols and *ortho*-diphenols were the strongest contributors to PC1, while flavonoids and ABTS activity primarily influenced PC2. These findings confirm that phenolic concentration, and specifically that of *ortho*-diphenols, is the principal driver of biochemical differentiation in these samples [46].

**Figure 3:**
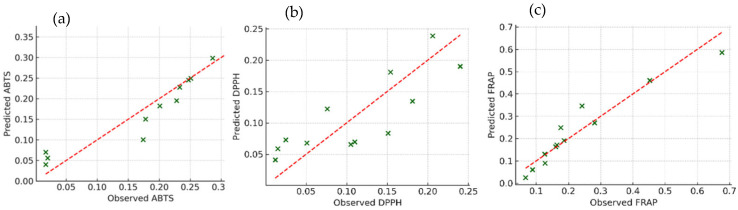
PLS regression of antioxidant capacity (ABTS, DPPH, FRAP) predicted by total phenols, *ortho*-diphenols, and flavonoids in olive oil by-products. Observed versus predicted values show strong correlations, highlighting the predictive power of phenolic traits across all assays. Each panel corresponds to one method: (**a**) ABTS, (**b**) DPPH, and (**c**) FRAP.

To validate and quantify these relationships, PLS regression was used to model the antioxidant activity based on phenolic markers. As shown in Figure 3, strong correlations were observed between the predicted and actual antioxidant capacity across all assays, with R^2^ values exceeding 0.9 for ABTS, DPPH, and FRAP. *Ortho*-diphenols consistently emerged as the best predictor of the antioxidant activity, followed by total phenols and flavonoids. These results agree with studies on other plant matrices, such as grape and cherry by-products, where PLS regression confirmed phenolic concentration as a reliable proxy for antioxidant function [47,48]. Figure 3 illustrates the relationship between observed and predicted antioxidant capacities (ABTS, DPPH, and FRAP), derived from PLS regression models based on phenolic content. Each green marker represents an individual sample, with the x-axis denoting the experimentally observed values and the y-axis representing the model’s predicted output. The dashed red line corresponds to the ideal 1:1 relationship, reflecting perfect agreement between prediction and observation. Data points closely aligned with this line indicate a high degree of predictive accuracy, whereas greater deviations suggest less precise estimations. Overall, the clustering of most samples near the diagonal line confirms the robustness of the PLS model in capturing the contribution of phenolic traits to antioxidant activity across assays. Among the three assays, the FRAP model demonstrated the highest predictive accuracy, followed by ABTS and, lastly, DPPH.

**Figure 4:**
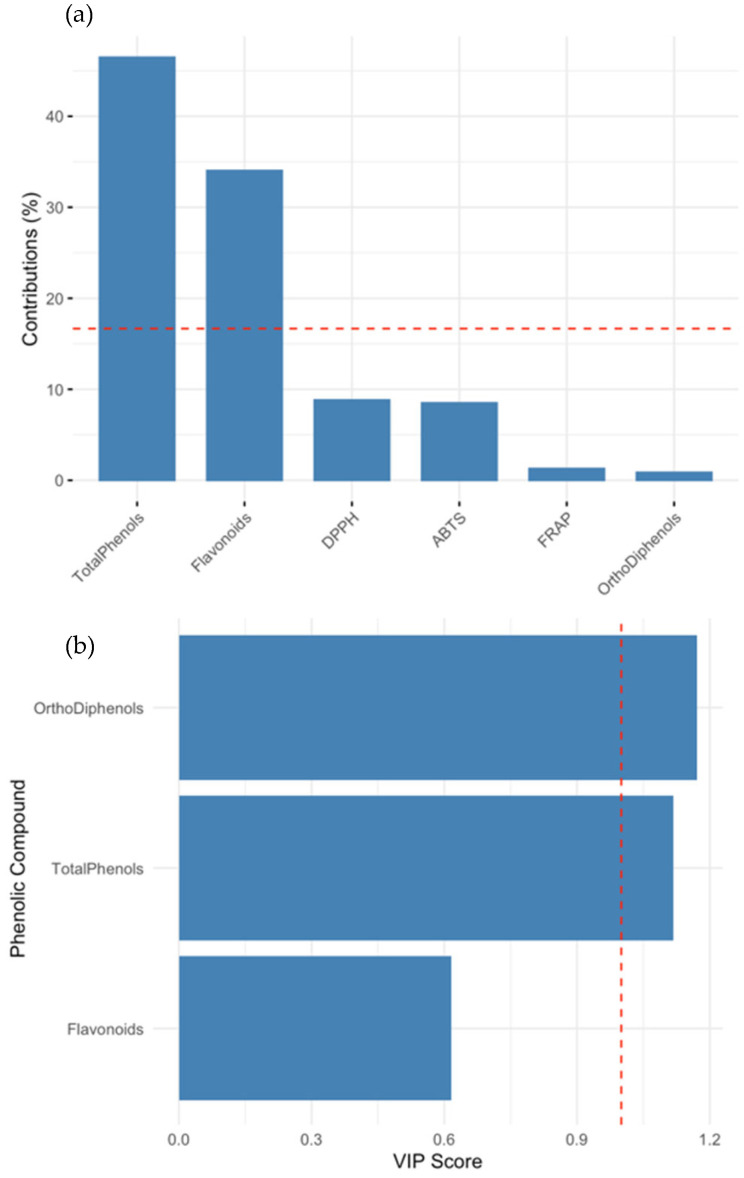
Comparison of variable importance across PCA and PLS models. (**a**) Contribution of phenolic and antioxidant markers to Principal Component 2 (Dim2) from PCA. Total phenols and flavonoids were the dominant contributors, suggesting that Dim2 captures phenolic variation more than antioxidant response. (**b**) Variable importance in projection (VIP) scores from PLS regression modeling ABTS antioxidant capacity. *Ortho*-diphenols and total phenols had VIP scores > 1, confirming their role as major predictors. The red dashed line indicates the VIP threshold of 1.

Figure 4 integrates the PCA and PLS findings to provide additional insights. In panel A, the contribution of phenolic variables to PCA Dim2 is shown, with total phenols and flavonoids emerging as the dominant contributors. Panel B displays the VIP scores derived from the ABTS-targeted PLS model. *Ortho*-diphenols and total phenols had VIP scores well above the commonly accepted threshold of 1, indicating their critical role in explaining the antioxidant behavior. ABTS and DPPH also contributed to the model, though to a lesser extent. These outcomes support the conclusion that *ortho*-diphenols are among the most potent antioxidants within the phenolic subclasses found in olive tissues. Their antioxidant efficacy stems from their catechol moiety, which enables efficient hydrogen donation, radical scavenging, and metal ion chelation. These structural features make them particularly effective at neutralizing reactive oxygen species. When compared to other subclasses, like flavonoids, *ortho*-diphenols often exhibit superior activity due to their ability to undergo redox cycling and stabilize radical intermediates. Flavonoids also contribute to antioxidant capacity, but their efficacy can be influenced by factors like glycosylation patterns and the presence of other functional groups. Thus, while both groups play vital roles, *ortho*-diphenols are generally more potent under oxidative stress conditions. Their antioxidant efficacy stems from their catechol moiety, which enables efficient hydrogen donation, radical scavenging, and metal ion chelation. Comparative studies suggest that *ortho*-diphenols exhibit higher reactivity toward peroxyl radicals than other subclasses, like monophenols or flavonoids, enhancing their biological utility in oxidative stress mitigation.

Overall, the multivariate analyses confirm that phenolic profiles are both tissue- and cultivar-dependent. Leaves and seeds emerge as the most promising sources of phenolic compounds, particularly in the ‘Cobrançosa’ and NI cultivars. The low antioxidant activity observed in stones, especially from ‘Galega’, is consistent with their known chemical composition, dominated by cellulose and lignin, which limits their potential for phenolic extraction. While phenolics appear to play a major role in the antioxidant capacity of olive by-products, further investigation is needed to clarify the contribution of other bioactive compounds, such as pigments (e.g., chlorophylls and carotenoids), which were not directly analyzed in this study. These findings provide a strong foundation for the targeted valorization of specific tissues and cultivars. The high antioxidant potency of phenolic-rich extracts suggests promising avenues for commercial applications in the food, cosmetic, and pharmaceutical sectors, where natural antioxidants are increasingly favored over synthetic alternatives. For example, phenolic-rich olive leaf extracts are already used as dietary supplements and natural preservatives, and there is an increasing interest in incorporating these into skincare formulations due to their anti-inflammatory and anti-aging properties. Clinically, hydroxytyrosol and oleuropein are being explored for their potential roles in cardiovascular protection and anti-cancer strategies, supported by regulatory frameworks that recognize certain phenolic compounds as bioactive ingredients with Generally Recognized as Safe (GRAS) status in many jurisdictions. For instance, leaves and branches may be selectively harvested for use in nutraceutical or cosmetic formulations, while seeds can be directed toward lipid-rich extract development. In contrast, stones may be more suitable for applications such as biomass energy or structural materials. This approach aligns with the circular economy principles by transforming agricultural waste into high-value bioresources, supporting both environmental sustainability and industrial innovation.

### 2.3. Identification of Individual Phenolic Compounds by High-Performance Liquid Chromatography (HPLC–PDA–MS)

To characterize the phenolic profiles of the leaves, stalks, seeds, and stones of the cultivars ‘Cobrançosa’, ‘Galega’, and NI, an analysis was carried out using high-performance liquid chromatography coupled with diode array detector (HPLC–DAD) and mass spectrometry (LC–MS). This methodology made it possible to identify the compounds based on retention time, absorption spectra, and mass-to-charge ratio (*m*/*z*). Significant differences in phenolic composition were observed between the different by-products. The main compounds identified included secoiridoids, lignans, flavonoids, simple phenols, phenylpropanoids, phenylethanoids, and coumarins. A summary of the bioactive compounds identified in the leaf, branch, seed, and stone extracts analyzed using MS techniques is reported in Table 3 and Table 4.

**Figure 5:**
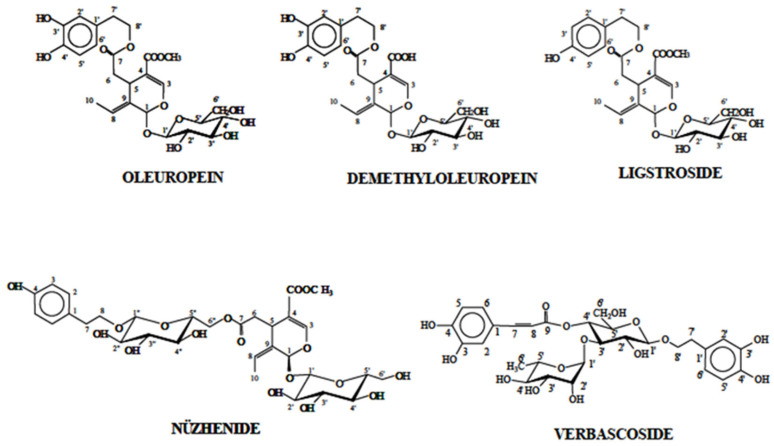
Chemical structures of secoiridoid derivatives and phenyl alcohols of olives.

The phenolic profiles of olive by-products from the cultivars ‘Cobrançosa’, ‘Galega’, and NI were comprehensively characterized by HPLC–DAD and LC–MS, highlighting significant compositional differences across leaves, branches, seeds, and stones. The leaves and branches showed a rich diversity of phenolic compounds, dominated by secoiridoids (Figure 5), flavonoids, phenylpropanoids, phenylethanoids, and lignans. This complex profile aligns with previous studies reporting high concentrations of oleuropein and its derivatives in leaves, as well as a variety of flavonoid glycosides, contributing to their bioactivity [49,50].

Secoiridoids, such as oleuropein diglucoside isomers (*m*/*z* 701) and their esterified forms, were the main compounds detected in leaves and branches, reinforcing their importance as key antioxidants in olive foliage [51,52]. The presence of methoxyoleuropein isomers (*m*/*z* 553) and hydroxyoleuropein (*m*/*z* 555), as well as a high molecular weight ligstroside derivative (*m*/*z* 1377.71) detected in the NI leaf extract, further exemplifies the structural complexity of these matrices, potentially influencing both stability and bioefficacy [53,54]. Flavonoids, like rutin (*m*/*z* 609), luteolin derivatives (*m*/*z* 447), and apigenin glucosides (*m*/*z* 431), were also abundant in these tissues, supporting their recognized contribution to anti-inflammatory and antioxidant effects [55,56].

In contrast, the seeds and stones exhibited a distinct phenolic profile, with a generally lower total phenolic content but a notable enrichment in lignans, particularly nuzhenide (*m*/*z* 685) and its esterified form nuzhenide 11-methyloleoside (*m*/*z* 701), consistent with other studies [57,58]. These lignans are known for their potent antioxidant and health-promoting properties, suggesting that seeds and stones may serve as valuable sources of these compounds. Secoiridoids were also detected in seeds and stones but mainly as minor or specific esterified forms, such as dihydrooleuropein isomers (*m*/*z* 541), oleuropein hexosides (*m*/*z* 701), and oleoside methyl esters (*m*/*z* 775), and in the case of ‘Galega’ stone extract, a high-mass compound tentatively identified as a complex secoiridoid derivative (*m*/*z* 1723.76). These findings are in agreement with the reports that the distribution and concentration of secoiridoids are tissue-specific within olive fruits [59,60].

Phenylpropanoids and phenylethanoids, such as verbascoside and decaffeoylverbascoside isomers (*m*/*z* 623), were present mainly in seeds and stones, but at lower levels than in leaves, which corresponds to the limited data available for these compounds in olive by-products [61,62]. The detection of epicatechin (*m*/*z* 289) in ‘Cobrançosa’ seeds and isoverbascoside in ‘Galega’ stones further confirms the complexity of the phenolic composition even in these less abundant matrices [63,64].

Overall, the phenolic composition clearly varies between olive by-products and cultivars, with leaves and branches being rich in diverse secoiridoids and flavonoids, while seeds and stones are characterized by higher lignan content and specific esterified secoiridoids. These findings underscore the potential of valorizing different olive waste fractions for tailored applications in nutraceutical, cosmetic, or food industries, fitting within sustainable circular economy models [65,66].

#### Structure–Activity Relationship and Correlation with Antioxidant Capacity

The antioxidant capacity of olive by-products, assessed through ABTS, DPPH, and FRAP assays, showed clear cultivar- and tissue-specific variations that align with the structural characteristics and abundance of the phenolic compounds identified.

Leaf extracts, particularly from the NI cultivar, exhibited the highest antioxidant activity across all three assays (ABTS: 0.323 ± 0.056; DPPH: 0.206 ± 0.009; and FRAP: 0.676 ± 0.036). This superior performance can be directly attributed to the presence of abundant and structurally potent secoiridoids, such as oleuropein diglucoside isomers and methoxyoleuropein, as well as flavonoids like rutin and luteolin glucoside isomers. These compounds contain multiple hydroxyl groups and conjugated double bonds, which enhance their radical scavenging activity by stabilizing unpaired electrons. Furthermore, the high FRAP value in NI leaves reflects the strong reducing capacity typical of compounds bearing catechol groups (e.g., luteolin derivatives).

In contrast, ‘Galega’ leaves presented significantly lower antioxidant values (ABTS: 0.201 ± 0.009; DPPH: 0.076 ± 0.002; and FRAP: 0.281 ± 0.007), which is consistent with the limited diversity and lower abundance of key secoiridoids and flavonoids observed in this cultivar.

Branch extracts, especially from ‘Cobrançosa’, also demonstrated considerable antioxidant potential (DPPH: 0.240 ± 0.025; FRAP: 0.242 ± 0.006), likely due to the presence of similar oleuropein derivatives, including diglucosides and esterified forms. The presence of flavonoid glycosides may further contribute to the antioxidant potential, particularly in DPPH, where hydrogen-donating ability is critical.

Seed extracts showed moderate antioxidant activity, with NI and ‘Galega’ seeds performing better in DPPH and ABTS, despite generally lower total phenolic content. This suggests the influence of specific compounds, such as nuzhenide, nuzhenide 11-methyloleoside, and epicatechin, which are structurally equipped with multiple phenolic hydroxyl groups, contributing to free radical scavenging. Interestingly, despite the low FRAP values in seeds, the presence of lignans like nuzhenide—less efficient as reductants but effective in radical quenching—may explain the relatively higher values in ABTS and DPPH compared to stones.

Stone extracts, particularly from NI and ‘Cobrançosa’, showed the lowest overall antioxidant activity across all assays. This correlates with the detection of fewer phenolic compounds and the dominance of highly esterified secoiridoids and lignans, such as the complex compound tentatively identified at *m*/*z* 1723.76, which, due to their large size and steric hindrance, may have reduced accessibility to active radical sites.

In summary, the antioxidant potential of the extracts is strongly influenced by the number and position of hydroxyl groups, degree of conjugation, and molecular size of the phenolic compounds. Secoiridoids with free phenolic groups and flavonoids with catechol structures were associated with the highest antioxidant activities. Conversely, highly glycosylated or esterified derivatives, while more stable, showed lower reactivity in redox-based assays like FRAP.

These findings support the relevance of structure–activity relationships in predicting the functional potential of phenolic-rich olive by-products and reinforce the importance of cultivar and tissue selection for targeted bioactivity.

### 2.4. Chlorophyll and Carotenoid Content

**Figure 6:**
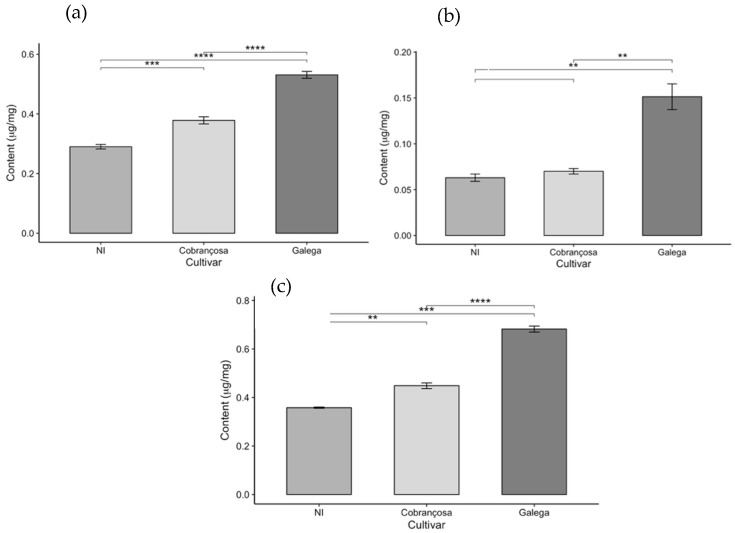
Chlorophyll content in olive leaves from three cultivars (NI, Cobrançosa, and Galega): (**a**) Chlorophyll a, (**b**) Chlorophyll b, and (**c**) Total chlorophyll (a + b), expressed as mean ± SD (*n* = 3). Statistical differences were assessed by pairwise *t*-tests. Asterisks indicate significance levels: *p* < 0.01 (**), *p* < 0.001 (***), and *p* < 0.0001 (****).

**Figure 7:**
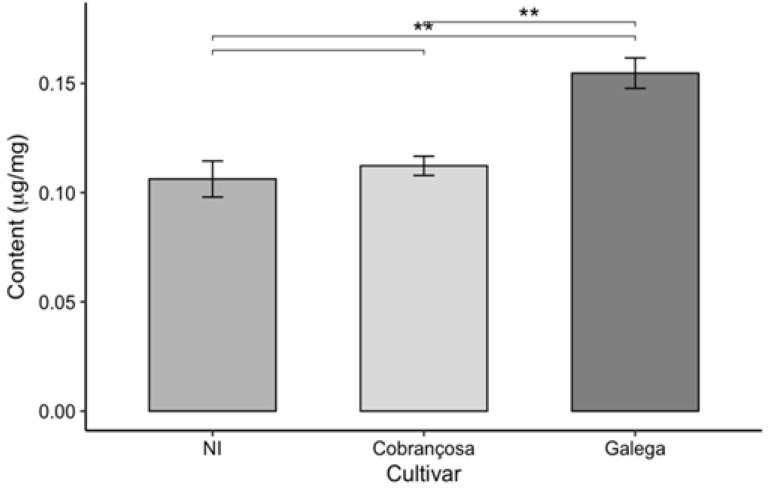
Carotenoid content in olive leaves from three cultivars (NI, Cobrançosa, and Galega), shown as mean ± SD (*n* = 3). Pairwise *t*-tests were used for statistical comparison. Asterisks indicate significance levels: *p* < 0.01 (**).

In addition to the quantification of total phenolic content, the assessment of antioxidant activity, and the individual identification of phenolic compounds by high-performance liquid chromatography (HPLC), the levels of chlorophyll a, chlorophyll b, total chlorophyll (a + b), and total carotenoids were also determined in the leaves of the three olive cultivars under study: an unidentified cultivar (NI), ‘Cobrançosa’, and ‘Galega’. The results obtained are presented in Figure 6 and Figure 7.

The analysis of photosynthetic pigment content in the leaves of the three olive cultivars (NI, ‘Cobrançosa’, and ‘Galega’) revealed significant differences among the cultivars, as it can in the above figures.

The ‘Galega’ cultivar stood out by exhibiting the highest levels of chlorophyll a, chlorophyll b, total chlorophyll (a + b), and carotenoids, with respective values of 0.531, 0.151, 0.682, and 0.155 µg/mg. These results highlight the strong photosynthetic capacity and bioactive potential of this cultivar’s leaves, reflected in their pigment composition. Consistent with these findings, Bahloul et al. [67] reported a chlorophyll a content like that of ‘Galega’ when analyzing various parameters—including chlorophyll a, chlorophyll b, and total chlorophyll—in three olive cultivars. However, the ‘Chemchali’ cultivar studied by these authors exhibited higher values for the other pigments than those found here, likely due to genetic differences among cultivars and the distinct environmental and agronomic conditions in which the plants were grown. Such variation is common in plant chemical composition studies, as genetic and environmental factors, cultivation practices, and harvest timing all significantly influence the pigment profiles.

In contrast, the NI cultivar showed the lowest pigment levels across all parameters evaluated—0.29, 0.063, 0.358, and 0.106 µg/mg for chlorophyll a, chlorophyll b, total chlorophyll, and carotenoids, respectively. The pigment levels measured in this study were lower than those reported for identified olive cultivars in the literature, such as in the work of Lorini et al. [68]. Nevertheless, the unidentified status of the NI variety limits direct comparison with named cultivars. These discrepancies may be attributed not only to genetic and environmental factors but also to differences in extraction and analytical methodologies used across studies.

The ‘Cobrançosa’ cultivar exhibited intermediate values for all analyzed parameters, positioning itself between the other two cultivars with levels of 0.378, 0.151, 0.529, and 0.155 µg/mg for chlorophyll a, chlorophyll b, total chlorophyll, and carotenoids, respectively. While there are limited data specifically quantifying pigments in this cultivar, Malheiro et al. [69] reported significantly lower chlorophyll a concentration (ranging from 0.0018 to 0.0029 µg/mg) in olive oil enriched with ‘Cobrançosa’ leaves, likely reflecting differences related to extraction methods and product matrices.

When comparing antioxidant activity to chlorophyll and carotenoid content, this study generally observed an inverse relationship; higher pigment levels corresponded to lower antioxidant activity in olive leaves. This finding contrasts with much of the scientific literature, where a positive correlation between these parameters is frequently reported. Ribas et al. [70] noted that leaf age can significantly influence chlorophyll and carotenoid content, as leaf coloration changes with aging, which may explain some of the observed results of this study.

Another plausible explanation is that the high antioxidant activity is not primarily driven by chlorophylls and carotenoids but rather by other phenolic compounds, such as flavonoids and *ortho*-diphenols, which are well known for their potent antioxidant effects. This hypothesis is supported by the data presented here, which highlight a strong presence of these phenolic compounds. Uğuz et al. [28] further corroborated this by showing that removing chlorophyll from olive leaf extracts did not diminish antioxidant activity, indicating phenolics as the main contributors to antioxidant effects. Nevertheless, chlorophylls may still play a role in cytotoxic properties, potentially contributing to anticancer effects, thus adding functional value to olive leaf extracts. These pigments (chlorophylls and carotenoids) may partly explain the antioxidant capacity observed in Section 2.2, highlighting their contribution alongside phenolic compounds.

In summary, the integrated analysis of pigment content and antioxidant activity reveals the biochemical complexity of olive leaves and underscores the importance of considering multiple genetic, environmental, and metabolic factors for the accurate valorization of these natural resources. A deeper understanding of pigment and phenolic profiles in different cultivars supports the development of efficient strategies in the food, pharmaceutical, and cosmetic industries, promoting sustainability and enhanced utilization of olive by-products.

## 3. Materials and Methods

### 3.1. Chemicals and Reagents

Aluminum chloride, sodium carbonate, sodium acetate, sodium nitrite, and TPTZ were obtained from Sigma–Aldrich (Steinheim, Germany). The Folin–Ciocalteu reagent and acetonitrile were obtained from Carlo Erba (Milan, Italy). ABTS•+, methanol, and formic acid were obtained from VWR Amresco (Fountain Parkway, Solon, EUA), 2,2-diphenyl-1-picrylhydrazyl (DPPH•) was obtained from Thermo Scientific (Waltham, MA, USA, EUA), and 6-hydroxy-2,5,7,8-tetramethylchromone-2-carboxylic acid (Trolox) was obtained from Sigma-Aldrich (Steinheim, Germany). Catechin and gallic acid were obtained from Sigma LifeScience. Trolox, potassium persulfate, and iron chloride were obtained from Aldrich Chemistry (Darmstadt, Alemanha). Hydrochloric acid was obtained from Merck Chemicals (Darmstadt, Germany). Sodium molibdate, sodium hydroxide, and aluminum chloride were obtained from Panreac Química S.L.U. (Barcelona, Spain). Ultrapure water was prepared using a Millipore (Burlington, MA, USA) water purification system.

### 3.2. Cultivar By-Product Samples

The olive by-products analyzed in this study—including leaves, branches, stones, and seeds—were collected during the 2024 harvest campaign from three different cultivars in an olive grove located in the Manteigas region. Sampling was conducted prior to the olive oil extraction process to avoid any influence from processing on the composition of the by-products. For each cultivar, a total of 9 independent samples were collected, corresponding to 3 trees per cultivar randomly selected within the olive grove. From each tree, 3 replicates of each by-product (leaf, branch, stone, seed) were sampled to account for intra-tree variability. This sampling design ensured a representative and statistically robust dataset for comparison between cultivars.

Sampling was performed during the same period for all cultivars, under similar environmental and phenological conditions, to minimize variation due to temporal factors. Among the cultivars, two were accurately identified as ‘Galega’ and ‘Cobrançosa,’ while the third cultivar remains unidentified due to the absence of clear morphological or genetic markers for precise classification.

Only healthy, undamaged plant material free of infections or physical defects was harvested to ensure sample integrity. Moreover, all samples were carefully collected from the same trees within each cultivar and from the exact same geographical location, thereby ensuring uniformity in soil type, microclimate, and other environmental variables. This careful sampling strategy minimized variability attributable to external factors, enhancing the reliability of comparative analyses between cultivars.

Following collection, the by-products were immediately freeze-dried using a Vir BenchTop Pro freeze dryer (SP Scientific, Warminster, PA, USA) to preserve their biochemical composition. The dried material was then ground into a fine powder using a blender and stored in a dark environment at room temperature until further analysis.

### 3.3. Phenolic Extract Preparation

**Figure 8:**
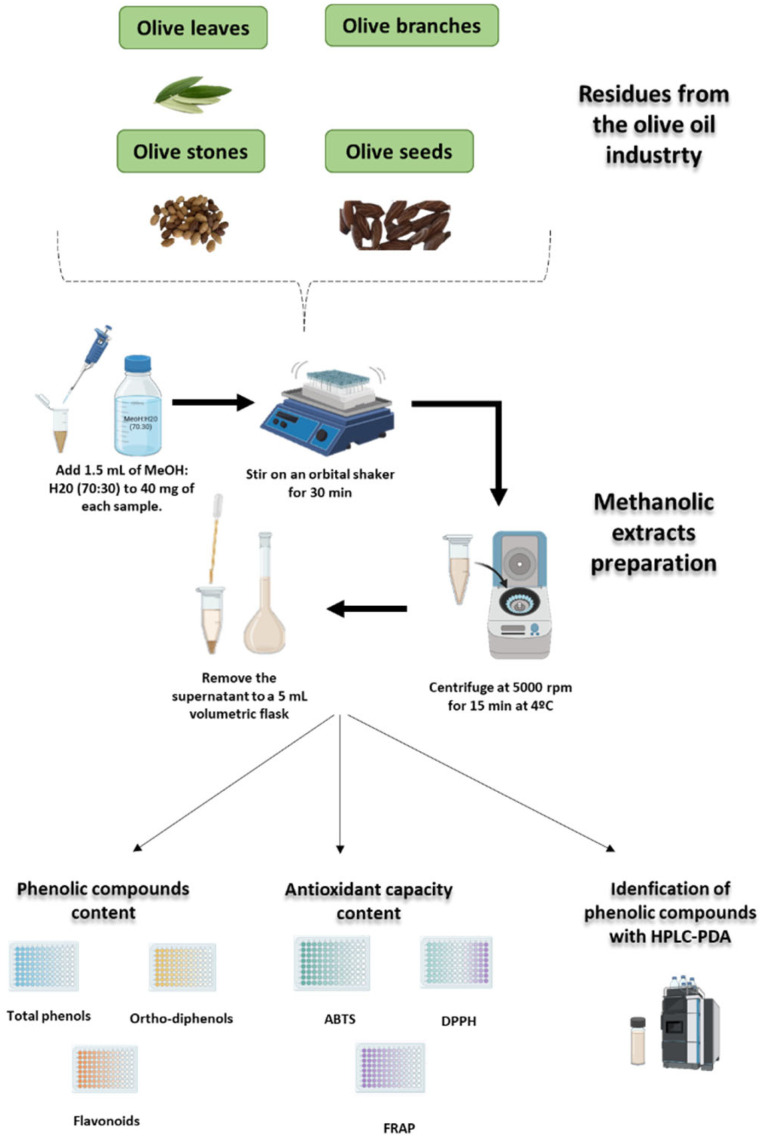
Schematic representation of the methodology used to extract and quantify polyphenols (total phenols, ortho-diphenols, and flavonoids), measure antioxidant capacity (FRAP, DPPH, and ABTS), and identify individual phenolic compounds by high-performance liquid chromatography in olive leaves, branches, seeds, and stones.

The phenolic extracts were prepared following the protocol described by Paié-Ribeiro et al. [7], with slight modifications. Briefly, 40 mg of each freeze-dried and ground sample was mixed with 1.5 mL of 70% (*v*/*v*) methanol solution and vortexed continuously for 30 min at room temperature. Subsequently, the mixture was centrifuged at 5000 rpm for 15 min at 4 °C using a Sigma centrifuge (Steinheim, Germany). The supernatant was carefully decanted from the solid residue, and the volume was adjusted to 5 mL. This extraction procedure was performed in triplicate for each sample, with the supernatants from each cycle combined to yield the final extract (see Figure 8).

### 3.4. Determination of Phenolic Content

The content of phenolic compounds was determined using methodologies previously described by [1,3] by quantifying total phenols, *ortho*-diphenols, and flavonoids. The analyses were carried out in triplicate (*n* = 3) in 96-well microplates, and the absorbances were measured using the Multiskan Go microplate reader (Thermo Fisher Scientific).

The total phenolic content was determined by the Folin–Ciocalteu spectrophotometric method, using gallic acid as a standard, and the results were expressed as mg of gallic acid per gram of dry weight (mg GA g^−1^ DW).

The flavonoid content was measured using the aluminum complex method, using catechin as a standard, with the results expressed in mg of catechin per gram of dry weight (mg CAT g^−1^ DW).

To quantify *ortho*-diphenols, the samples were treated with sodium molybdate (50 g L^−1^), and the absorbance was measured at 375 nm, with gallic acid as the standard. The results were expressed as mg of gallic acid per gram of dry weight (mg GA g^−1^ DW).

### 3.5. Determination of Antioxidant Capacity

The antioxidant capacity was measured using the following methodologies previously described by Monteiro et al. [2] and Brito et al. [3]: 2,2-diphenyl-1-1-picrylhydrazyl (DPPH), Fe^3+^-reducing capacity in Fe^2+^ (FRAP), and free radical scavenging activity of the cation radical (ABTS). The analyses were carried out in triplicate (*n* = 3) in 96-well microplates, with absorbance measurements taken on Multiskan Go equipment (Thermo Fisher Scientific).

The ABTS test was carried out by reacting ABTS+ and the sample or standard, and the absorbance readings were taken at 734 nm.

For the DPPH method, the DPPH+ solution was added to a Trolox standard or sample, and the absorbances were read at 520 nm. The results of the ABTS and DPPH methods were expressed as mmol of Trolox per gram of dry sample (mmol Trolox g 1 DW).

The FRAP assay was carried out by reacting FRAP with the sample or standard. The absorbances were read at a wavelength of 593 nm.

### 3.6. Analysis of Individual Phenolic Compounds by High-Performance Liquid Chromatography

The powdered samples (40 mg) were mixed with 1.5 mL of methanol/water (70:30), according to Paié-Ribeiro et al. [4], with some changes. The samples were then shaken on an orbital shaker (GFL, Bad Oldesloe, Germany) for 30 min and centrifuged in a centrifuge (Sigma, Osterode am Harz, Germany) for 15 min. The resulting extracts were filtered through a 0.22 μm PVDF filter (Millex HV13, Millipore, Bedford, MA, USA) and stored in vials.

The chromatographic separation of the phenolic compounds present in the analytical extracts was carried out according to the procedure described by Paié-Ribeiro et al. [4] using a Luna C18 column (250.0 × 4.6 mm, particle size 5.0 μm, Phenomenex, Macclesfield, UK) and an Agilent 1100 series HPLC equipped with diode array detector and serial mass detector (Agilent Technologies, Waldbronn, Germany). The HPLC consisted of a binary pump (model G1312A), an autosampler (model G1313A), a degasser (model G1322A), a photodiode array (PDA) detector (model G1315B), and an ion trap spectrometer (model G2445A) equipped with an electrospray ionization interface and controlled by LCMSD software (v. 4.1, Agilent Technologies). The mobile phases used were deionized water/formic acid (99.0:1.0, *v*/*v*) (solvent A) and acetonitrile/formic acid (99.0:1.0, *v*/*v*) (solvent B). The spectra of all the peaks were detected in the 200 to 600 nm range, and chromatograms were recorded at 280 nm for proanthocyanidins, 330 nm for phenolic acids and stilbenes, 360 nm for flavonols, and 520 nm for anthocyanins. The HPLC–PDA–ESI/MSn analyses were carried out using ChemStation software (v. 08.03, Agilent Technologies). Mass spectrometry data was acquired in negative (proanthocyanidins, phenolic acids, secoiridoids, flavonols, and flavons) ionization modes. The phenolic compounds were identified by examining the retention time (min), the parent ions, and the databases and descriptions available in the literature. The phenolic compounds were characterized and quantified by PDA chromatograms observed at the different wavelengths described above for the different phenolic classes, using calibration curves prepared daily with catechin (proanthocyanidins), chlorogenic acid (phenolic acids), resveratrol (stilbenes), quercetin-3-O-glucoside (flavonols), and cyanidin-3-O-glucoside (anthocyanins).

### 3.7. Quantitative Analysis of Chlorophylls and Carotenoids

Chlorophyll and carotenoid contents were quantified to evaluate the photosynthetic pigment profiles of olive leaves, providing additional biochemical markers associated with cultivar-specific metabolic traits and valorization potential.

The method of Barros et al. [5] was followed. Pigments were extracted from 5 mg of each sample using 80% acetone, incubated for 24 h at 4 °C, and centrifuged at 5000 rpm for 10 min. Absorbance was measured at 663.2 nm (chlorophyll a), 646.6 nm (chlorophyll b), and 470 nm (carotenoids) using a Thermo Electron spectrophotometer. Results were expressed in µg/mg dry weight as mean ± SD of three replicates.

Chlorophyll a (Ca) = 12.25 × A_663_ − 2.55 × A_646_ [μg/mL] (1)

Chlorophyll b (Cb) = 20.31 × A_646_ − 4.91 × A_663_ [μg/mL] (2)

Total chlorophyll (a + b) = 17.76 × A_646_ + 7.34 × A_663_ [μg/mL] (3)

Carotenoids = (1000 × A_470_ − 1.82 × Ca − 85.02 × Cb)/198 [μg/mL] (4)

(1) A_663_: Absorbance at 663 nm (peak for chlorophyll a); A_646_: Absorbance at 646 nm (peak for chlorophyll b). This formula estimates the amount of chlorophyll a by correcting for overlap with chlorophyll b.
(2) This formula estimates chlorophyll b by correcting for the contribution of chlorophyll a.
(3) This equation combines both chlorophylls a and b to estimate total chlorophyll content.
(4) A_470_: Absorbance at 470 nm (characteristic for carotenoids). This equation removes interference from chlorophylls a and b to estimate true carotenoid content.

## 4. Conclusions

The present study contributes to the ongoing efforts to promote a circular economy within the agri-food sector by exploring the valorization of olive oil by-products as potential sources of bioactive compounds. Through detailed chemical characterization, it was possible to confirm that leaves and seeds—particularly from the ‘Cobrançosa’ and NI cultivars—stand out for their high content of phenolic compounds and marked antioxidant capacity. Multivariate analyses, including PCA and PLS regression, reinforced these findings by identifying total phenols and *ortho*-diphenols as the variables most strongly associated with antioxidant performance. These compounds, along with oleuropein, hydroxytyrosol, and verbascoside, appear to be the main contributors to the observed bioactivity. Although stones revealed limited potential due to their low phenolic content, branches—especially from ‘Cobrançosa’—presented a more favorable profile, highlighting an underutilized resource with possible functional value. Additionally, the inverse correlation found between pigment content and antioxidant activity supports the predominance of phenolic compounds as key drivers of bioactivity in these matrices. Altogether, the results underline the potential of olive by-products, particularly leaves and branches, as valuable sources of natural antioxidants for application in nutraceutical, food, and pharmaceutical formulations. Their recovery and reuse contribute not only to the reduction of agro-industrial waste but also to the achievement of European sustainability goals for resource efficiency and innovation.

## Figures and Tables

**Table 1:** Total phenol (mg gallic acid equivalents [GAE]/g dry weight [DW]), ortho-diphenol (mg GAE/g DW), and flavonoid content (mg catechin equivalents [CAT]/g DW) of the leaf, seed, stone, and branch from ‘Cobrançosa’, ‘Galega’, and NI cultivars. Data are presented as mean ± standard deviation (SD). Different letters in the same column indicate significant differences between species (*p* < 0.05), according to one-way ANOVA, followed by Tukey’s post hoc test.

		Phenolic Content		
		Total Phenols	*Ortho*-Diphenols	Flavonoids
Leaf	NI	66.147 ± 4.872 ^a^	89.301 ± 4.463 ^a^	54.297 ± 5.073 ^a^
	‘Cobrançosa’	55.117 ± 1.043 ^b^	78.177 ± 0.681 ^b^	39.119 ± 1.321 ^b^
	‘Galega’	32.878 ± 0.961 ^c^	45.627 ± 0.819 ^c^	28.659 ± 1.834 ^c^
Seed	NI	78.032 ± 2.087 ^b^	42.857 ± 0.158 ^a^	10.368 ± 2.533 ^a^
	‘Cobrançosa’	86.116 ± 0.336 ^a^	36.028 ± 0.553 ^b^	10.849 ± 0.641 ^a^
	‘Galega’	75.674 ± 0.872 ^b^	35.613 ± 0.490 ^b^	10.782 ± 1.376 ^a^
Stone	NI	9.310 ± 0.489 ^b^	9.389 ± 0.446 ^b^	17.352 ± 0.243 ^a^
	‘Cobrançosa’	11.867 ± 1.498 ^a^	11.696 ± 1.351 ^a^	21.180 ± 2.706 ^a^
	‘Galega’	6.534 ± 0.614 ^c^	6.882 ± 0.545 ^c^	12.477 ± 0.849 ^b^
Branch	NI	19.406 ± 0.706 ^c^	19.245 ± 2.165 ^b^	22.571 ± 2.385 ^c^
	‘Cobrançosa’	35.118 ± 0.633 ^a^	35.209 ± 2.396 ^a^	51.715 ± 5.389 ^a^
	‘Galega’	24.753 ± 1.289 ^b^	32.406 ± 2.408 ^a^	35.521 ± 1.556 ^b^

**Table 2:** Antioxidant capacity (mmol Trolox/g DW) of the leaves, seeds, stones, and branches from ‘Cobrançosa’, ‘Galega’, and NI cultivars. Data are presented as mean ± SD. Different letters in the same column correspond to significant differences between species (*p* < 0.05) determined using an ANOVA, followed by a post hoc Tukey’s test.

		Antioxidant Capacity		
		ABTS	DPPH	FRAP
Leaf	NI	0.323 ± 0.056 ^a^	0.206 ± 0.009 ^a^	0.676 ± 0.036 ^a^
	‘Cobrançosa’	0.286 ± 0.007 ^a^	0.154 ± 0.007 ^b^	0.452 ± 0.004 ^b^
	‘Galega’	0.201 ± 0.009 ^b^	0.076 ± 0.002 ^c^	0.281 ± 0.007 ^c^
Seed	NI	0.247 ± 0.005 ^a^	0.110 ± 0.003 ^a^	0.186 ± 0.003 ^a^
	‘Cobrançosa’	0.251 ± 0.003 ^a^	0.051 ± 0.043 ^a^	0.164 ± 0.002 ^b^
	‘Galega’	0.233 ± 0.003 ^b^	0.105 ± 0.025 ^a^	0.160 ± 0.003 ^b^
Stone	NI	0.020 ± 0.001 ^a^	0.015 ± 0.003 ^ab^	0.088 ± 0.016 ^b^
	‘Cobrançosa’	0.017 ± 0.001 ^b^	0.025 ± 0.001 ^a^	0.127 ± 0.012 ^a^
	‘Galega’	0.017 ± 0.007 ^ab^	0.012 ± 0.007 ^b^	0.067 ± 0.007 ^b^
Branch	NI	0.174 ± 0.048 ^a^	0.151 ± 0.041 ^b^	0.126 ± 0.021 ^c^
	‘Cobrançosa’	0.228 ± 0.009 ^a^	0.240 ± 0.025 ^a^	0.242 ± 0.006 ^a^
	‘Galega’	0.178 ± 0.003 ^a^	0.181 ± 0.011 ^ab^	0.176 ± 0.006 ^b^

**Table 3:** Identification of phenolic compounds present in olive leaf and branch extracts by HPLC–PDA–MS. RT: retention time; N.D.: not detected.

Compound Id	RT	λ (UV)	*m*/*z*	*m*/*z*		Leaf	
	(min)	(nm)	[M − H]^−^	Fragments	NI	‘Cobrançosa’	‘Galega’
**dihydro oleuropein**	1.13	360	541	419, 305, 289	X	X	N.D.
					N.D.		
**oleuropein diglucoside isomer 1**	12.06	360	701	607, 577, 417, 269	N.D.	X	X
**oleuropein diglucoside isomer 2**	12.22	360	701	607, 577, 269	N.D.	N.D.	X
**oleuropein diglucoside isomer 3**	12.34	360	701	607, 577	N.D.	x	N.D.
**oleuropein diglucoside isomer 4**	12.38	360	701	607, 431, 285	X	N.D.	N.D.
**Oleuropein diglucoside isomer 5**	12.58	360	701	607, 431	X	N.D.	x
**Ligstroside derivative**	12.65	360	1377.71	1125, 447, 285	X	N.D.	N.D.
**2″-methoxyoleuropein isomer 1**	13.15	360	553	447, 285	N.D.	X	N.D.
**2″-methoxyoleuropein isomer 2**	13.47	360	553	447, 285	X	N.D.	N.D.
**hydroxyoleuropein**	20.07	360	555	299	N.D.	N.D.	x
**rutin**	10.79	360	609	-	X	X	N.D.
**luteolin glucoside isomer 1**	11.39	360	447	285	N.D.	N.D.	x
					N.D.		
**hydroxytyrosol hexose isomer 2**	1.49	360	665.50	315	N.D.	N.D.	X
**verbascoside isomer 1**	11.04	36	623	463, 447	X	N.D.	N.D.
**verbascoside isomer 2**	11.18	360	623		N.D.	N.D.	X
**tyrosol glucoside**	20.06	360	299		N.D.	N.D.	X

**Table 4:** Identification of phenolic compounds present in olive seed and stone extracts by HPLC–PDA–MS. RT: retention time; N.D.: not detected.

Compound Id	RT	λ (UV)	*m*/*z*	*m*/*z*		Seeds			Stones	
	(min)	(nm)	[M − H]^−^	Fragments	NI	‘Cobrançosa’	‘Galega’	NI	‘Cobrançosa’	‘Galega’
**dihydro oleuropein isomer 1**	1.15	320	541	289, 159	N.D.	N.D.	N.D.	x	x	x
					N.D.					
**dihydro oleuropein isomer 2**	1.87	320	541	191, 129	N.D.	N.D.	N.D.	x	N.D.	N.D.
**dihydro oleuropein isomer 3**	2.17	320	541	191, 153	N.D.	N.D.	N.D.	N.D.	N.D.	x
					N.D.					
**oleuropein diglucoside isomer 1**	10.89	280	864	794, 701, 453	N.D.	x	x	N.D.	N.D.	N.D.
**oloseoside-11-methyloleoside**	11.87	280	775	685, 623, 523	x	N.D.	x	N.D.	N.D.	N.D.
**oleuropein hexoside isomer 1**	11.88	320	701	623, 543	N.D.	N.D.	N.D.	N.D.	N.D.	x
**oleuropein hexoside isomer 2**	12.31	320	701	685, 577, 453	N.D.	N.D.	N.D.	N.D.	x	N.D.
**6′-*O*-[(2E)-2.6-dimethyl-8-hydroxy-2-octenoyloxy]-secologanoside**	13.45	280	1723.76	557, 373	N.D.	N.D.	N.D.	N.D.	N.D.	X
					x					
					N.D.					
**hydroxyoleuropein**	22.67	320	555	433, 311	N.D.	N.D.	N.D.	x	N.D.	N.D.
**nuzhenide**	11.69	280	685	653, 523	x	x	x	N.D.	N.D.	N.D.
**nuzhenide 11-methyloleoside**	14.20	320	701	623, 461	N.D.	N.D.	N.D.	x	N.D.	x
					N.D.					
					N.D.					
**decaffeoylverbascoside isomer 1**	1.95	280	623	461	x	X	N.D.	N.D.	N.D.	N.D.
**decaffeoylverbascoside isomer 2**	2.36	280	623	461, 431	x	x	N.D.	N.D.	N.D.	N.D.
**epicatechin**	1.13	280	289	159, 131	N.D.	x	x	N.D.	N.D.	N.D.
					N.D.					
**apigenin 7-O-glucoside**	2.62	320	431	311, 151	N.D.	N.D.	N.D.	x	N.D.	N.D.
					N.D.					
**verbascoside**	11.12	320	623	505	N.D.	N.D.	N.D.	x	x	x
**isoverbascoside**	11.88	320	623	543	N.D.	N.D.	N.D.	N.D.	N.D.	x

## Data Availability

The original contributions presented in this study are included in the article. Further inquiries can be directed to the corresponding author.
